# Using spectral continuity to extract breathing rate from heart rate and its applications in sleep physiology

**DOI:** 10.3389/fphys.2024.1446868

**Published:** 2024-08-02

**Authors:** Gregoris A. Orphanides, Christodoulos Karittevlis, Lujain Alsadder, Andreas A. Ioannides

**Affiliations:** ^1^ Laboratory for Human Brain Dynamics, AAI Scientific Cultural Services Ltd., Nicosia, Cyprus; ^2^ Barts and The London School of Medicine and Dentistry, Queen Mary University of London, London, United Kingdom

**Keywords:** scEDR, ECG derived respiration (EDR), REM0, sleep staging, sleep autonomic system, breathing

## Abstract

**Introduction:** ECG Derived Respiration (EDR) are a set of methods used for extracting the breathing rate from the Electrocardiogram (ECG). Recent studies revealed a tight connection between breathing rate and more specifically the breathing patterns during sleep and several related pathologies. Yet, while breathing rate and more specifically the breathing pattern is recognised as a vital sign it is less employed than Electroencephalography (EEG) and heart rate in sleep and polysomnography studies.

**Methods:** This study utilised open-access data from the ISRUC sleep database to test a novel spectral-based EDR technique (scEDR). In contrast to previous approaches, the novel method emphasizes spectral continuity and not only the power of the different spectral peaks. scEDR is then compared against a more widely used spectral EDR method that selects the frequency with the highest power as the respiratory frequency (Max Power EDR).

**Results:** scEDR yielded improved performance against the more widely used Max Power EDR in terms of accuracy across all sleep stages and the whole sleep. This study further explores the breathing rate across sleep stages, providing evidence in support of a putative sleep stage "REM0" which was previously proposed based on analysis of the Heart Rate Variability (HRV) but not yet widely discussed. Most importantly, this study observes that the frequency distribution of the heart rate during REM0 is closer to REM than other NREM periods even though most of REM0 was previously classified as NREM sleep by sleep experts following either the original or revised sleep staging criteria.

**Discussion:** Based on the results of the analysis, this study proposes scEDR as a potential low-cost and non-invasive method for extracting the breathing rate using the heart rate during sleep with further studies required to validate its accuracy in awake subjects. In this study, the autonomic balance across different sleep stages, including REM0, was examined using HRV as a metric. The results suggest that sympathetic activity decreases as sleep progresses to NREM3 until it reaches a level similar to the awake state in REM through a transition from REM0.

## 1 Introduction

Vital signs are measurements that most closely reflect the patient’s internal environment, traditionally including the body’s temperature, blood pressure, respiratory, and Heart Rate (HR) (A list of all abbreviations can be found at Table 1) (Sapra et al., 2023). Even though studies have shown that increases in the breathing rate can indicate critical illness before other vital signs show any change (Nicolò et al., 2020; Chourpiliadis and Bhardwaj, 2022), it is often overlooked as it is especially hard to measure in the clinical setting. A recent study has also established a relationship between mortality from cardiovascular disease and nocturnal respiration rate (Baumert et al., 2019). Hence, there is a need for a non-invasive and accurate method to measure how breathing arises, for example, when investigating sleep disorders and apnea.

**TABLE 1:** Abbreviations used in the paper.

Abbreviation	
TFA	Time-frequency analysis
HR	Heart Rate
IP	Immediate Precursor
LL	Long Lead
mPRF	medial Pontine Reticular Formation
LGN	Lateral Geniculate Nucleus
HRV	HR variability
RSA	Respiratory Sinus Arrhythmia
OSA	Obstructive Sleep Apnea
ECG	Electrocardiogram
PSG	Polysomnography
REM	Rapid Eye Movement
NREM	Non-Rapid Eye Movement
EDR	ECG Derived Respiration
scEDR	Spectral Continuity ECG Derived Respiration
PPG	Photoplethysmography
VLF	Very Low Frequency (0.004Hz–0.05 Hz)
LF	Low Frequency (0.05Hz–0.15 Hz)
HF	High Frequency (0.15Hz–0.40 Hz)
SD	Standard Deviation
PGO	Ponto-Geniculo-Occipital
KCm	K-Complex multiple
GSR	Galvanic Skin Resistance
ATP	Adenosine Triphosphate
AMP	Adenosine Monophosphate

With the rise of obesity in the Western world, the prevalence of sleep apnea has become increasingly concerning, with estimates that 70% of obese patients have a high risk of developing moderate to severe obstructive sleep apnea (Ribeiro et al., 2020). Currently, sleep apnea is classified into two main types: obstructive sleep apnea (OSA) and central sleep apnea (CSA). In OSA, the airway is blocked due to muscle relaxation, while in CSA, the brain stops signaling the respiratory muscles to contract (Rana and Sankari, 2023). The current gold standard for the diagnosis of sleep apnea is sleep polysomnography, which has limited availability. In the case of sleep apnea diagnosis, clinical practice relies heavily on invasive flow meters (Laratta et al., 2017). The following paragraphs, discuss how a deeper understanding of the role and interaction of cardiovascular and breathing mechanisms during wakefulness and various sleep stages has broader implications for understanding normal sleep and its disorders.

Sleep staging was introduced in the late 1960s (Rechtschaffen and Kales, 1968) and was based mainly on EEG, alongside EMG and EOG measures to classify sleep into 7 distinct sleep stages based on the hallmarks of sleep present in each 30-s trial. Sleep staging was a tremendous advance, but still left some of the sleep as undefined, either because of noise in the measurements, or because the 30 s segments contained hallmarks (characteristics) belonging to more than one sleep stage. In 2007, new guidelines were introduced that combined NREM3 and NREM4 to a single new NREM3 sleep stage. Numerous additional instructions and criteria for classifying sleep stages based on the percentage of hallmarks present in each 30 s trial (Silber et al., 2007) were introduced. Neural networks have also been utilized in automatic sleep classification using either single EEG channels or by combining EEG with EOG at different weights to better reflect human expert classification (Jia et al., 2022). In recent years, methods have arisen that allow the transfer of knowledge from more complex neural networks to lightweight ones, allowing small devices with limited processing power to perform accurate automatic sleep classification (Liang et al., 2023).

At the same time there were numerous suggestions for elaboration of the sleep staging of early sleep, which were recently summarized (Biabani et al., 2023). This review states that “sleep onset bears a complex pattern associated with a multitude of behavioural and physiological markers and remains poorly understood”, with further adding that sleep onset “has fluctuating and ill-defined boundaries”. The review concludes with a plea for advances because “there is a recognized need for an international consensus on what constitutes a true sleep onset”. Furthermore, studies showing two distinct substates within REM inspired the division of REM into microstates (Wehrle et al., 2007). The case for separating REM into tonic and phasic sleep was strongly supported by Simor and colleagues based on the presence of eye movements (phasic REM) or absence of eye movements (tonic REM) (Simor et al., 2016). While additionally, showing that sensorimotor activity was mainly present during phasic REM followed by frontoparietal activity likely linked to environmental awareness (Simor et al., 2018). The same team further showed that alpha and beta activity was present during tonic REM while gamma activity was present during phasic REM (Simor et al., 2019) with the series of papers concluding that phasic and tonic REM microstates would facilitate the understanding of the mechanisms and functions of REM sleep in healthy and pathological conditions (Simor et al., 2020).

Simultaneously, with the early human sleep studies preparing the ground for sleep staging, work with animals was examining the role of the brainstem in sleep. Specifically, prominent propagating waves apparently originating in the brainstem during and around REM were identified, originally in the pons, lateral geniculate nucleus (LGN) and occipital lobe of cats and labelled as Ponto-Geniculo-Occipital (PGO) waves (Jouvet et al., 1959). In the last 4 decades of the 20th century sleep research on humans and animals proceeded in rather separate ways. Human studies helped establish sleep medicine, while animal research slowly unraveled some of the mysteries of PGO wave generation, distinguishing an immediate precursor (IP) and a long lead (LL) generator preceding the onset of first propagating component of the PGO wave in the LGN. The IP was located in midbrain/pons peribrachial region (Mccarley et al., 1978). While the LL generator was found in the medial pontine reticular formation (mPRF) (McCarley and Ito, 1983). What is remarkable during this period was that although the fact that REM sleep is associated “with significant perturbations in autonomic nervous system” was known for humans even before sleep stages were established (Snyder et al., 1964) its significance was realized much later (Rowe et al., 1999).

Early in this century, the advent of deep brain stimulation provided access to the electrophysiology of subcortical structures and produced evidence consistent with the presence of PGO waves in humans during and before REM, roughly corresponding to the animal generators of the IP and LL responses. Firstly, in the pontomesencephalic tegmentum corresponding roughly to halfway between the animal IP and LL generators, which were only incompletely associated with eye movements, and were followed by characteristic cortical potentials (Lim et al., 2007). Secondly, evidence for human PGO-like waves in the sub-thalamic nucleus was found, occurring typically before and during the bursts of rapid eye movements (Fernández-Mendoza et al., 2009). In the last 2 years, two attempts have been made to synthesize the work described in the last paragraphs supported by new data and analysis. The most recent synthesis (Bueno-Junior et al., 2023) is based on a study of the temporal structure of REM sleep in mice and humans. They point out that at the moment “There is no consensus on how to subdivide REM, both in terms of time binning and scoring criteria”. They propose that respiration rate combined with oculomotor activity could be a practical unifying measure of REM structure in humans and laboratory animals, as opposed to signals that are more accessible only in the laboratory, such as theta oscillations and P-waves (the pontine component that is often the term used instead of PGO waves in rodent studies).

The conclusion from the review of sleep staging evolution is that there are good reasons for including in the sleep staging criteria that now rely almost exclusively on the brain activity, criteria depending on the two other organs that are known to be linked to brain activity and influence sleep, the heart and the respiration. The introduction of REM0 was a direct consequence of adding the heart rate variance (Ioannides et al., 2022). The addition of measures of the activity of the respiratory system would be greatly facilitated by the availability of a fast, efficient and easy to use method for monitoring breathing, and this is exactly what we propose in this work. REM0 was suggested by (Ioannides et al., 2022) and is a period where slow waves are present in the EEG similar to NREM2 and NREM3, but it also has eye movements and heart rate surges (HRS) normally associated with REM sleep (Žemaityt et al., 1986; Penzel et al., 2003). The key distinction is that in REM0, HRS ride on an infra-slow frequency (0.05–0.10H) and are coupled with both large EEG graphoelements (K-complex multiples) and eye movements. Even though, splitting REM into substages mainly phasic and tonic REM based on the presence of eye movements is generally accepted (Simor et al., 2016; Simor et al., 2020), REM0 tends to be reclassified from periods not previously scored as REM which in conjunction with its mixed NREM and REM nature, place it as a putative new sleep stage.

The paragraphs above provide evidence supporting the need for a non-invasive and accurate method to measure the breathing rate, highlighting its importance as a measure with both theoretical and clinical applications. Such a low-cost and non-invasive indicator has applications in sleep research and very likely sleep medicine especially sleep apnea, where it can provide economically viable solutions to increase the rate of diagnosis or at least provide an early indication for high-risk patients. A possible means to monitor the breathing rate in both awake and sleep states appears to be the heart rate with many ECG Derived Respiration (EDR) methods already being proposed (Varon et al., 2020a). It is known that respiration can influence the heart rate by the effect of the vagal tone on pacemaker cells as commonly seen in Respiratory Sinus Arrhythmia (RSA) (Yasuma and Hayano, 2004), which results in an increase in the heart rate during inhalation and *vice versa*. This physiological rhythm modulation adds a specific frequency feature to the heart rate that can be isolated and quantified using frequency decomposition methods as described in (Sohrt-Petersen, 2014). The RSA spectral perturbation appears best as a low-frequency component that can be disentangled from the other components by previous EDR techniques. Previous studies have criticized frequency-based EDR methods because many frequency components arise from non-breathing-related means (Varon et al., 2020a). The methodology proposed in this study makes use of the observation that the presence of other spectral perturbations in the same frequency range are usually relatively short events appearing intermittently; in contrast, the ones due to RSA are present all the time. Therefore, the novel method can disentangle the true breathing rate from other slow components by identifying the one component that smoothly runs over long periods and hence, termed as spectral continuity EDR (scEDR). This study demonstrates that the correct selection of frequency components emerges naturally as the one with the most principled continuity in the range of frequencies relevant to breathing. The identification of the specific frequency component related to breathing makes it possible to reconstruct a breathing-related signal reliably from the heart rate, even in most of the periods when other frequencies in the extended band around the true breathing rate frequency are present. An important aim of this work is to explore how stable and accurate an estimate of breathing rate from the HR can scEDR provide, while not identifying advantages in addressing other specific research questions and potential clinical applications, which will be addressed in future studies.

This paper, firstly defines the novel scEDR method and then proceeds to test it using data from the open-access ISRUC-Sleep whole night sleep polysomnography (PSG) dataset as described in (Khalighi et al., 2016). For this paper, data from ten (10) healthy subjects (subgroup III of the ISRUC database) is used to validate the method. Additionally, eighteen (18) patients suffering from sleep apnea from subgroup I of the ISRUC database were used to further validate scEDR method. The data include breathing rate extracted from pressure flow meters, the gold standard for measuring breathing rate, which was used to validate scEDR results. The paper then explores the normal breathing patterns across different sleep stages using the latest five-stage sleep staging (Iber, 2007; Silber et al., 2007). While later, augmenting the investigation of sleep stages by including in the analysis the newly suggested but not widely discussed REM0, as a putative new sleep stage (Ioannides et al., 2022).

## 2 Materials and methods

### 2.1 Data used experimental dataset

Subgroup I and III of the ISRUC-Sleep whole night polysomnography (PSG) open-access dataset (Khalighi et al., 2016) was used in this work. Subgroup III consists of 10 healthy subjects’ single whole night sleep PSG recordings. This dataset provides recordings from a plethora of sensors; most importantly for this paper, an ECG electrode and pressure-based flow meters. This collection of data allows for validation of the breathing rate extracted using scEDR. Additionally, pulse oximeter and microphone channels are integrated that can be used for testing for OSA. More details about the sensors used and the subgroups can be found in Tables 3, 4 respectively found at (Khalighi et al., 2016). The first part of the analysis applies scEDR to the data from subgroup III to extract the breathing rate and to compare the results with the gold standard analysis using the flow meter data combined with the previously described spectral EDR method, which selects the frequency with the highest power within the breathing frequency. The follow-up analysis quantifies patterns of spectral power distribution across the normal sleep cycle, with and without the definition of REM0 periods. Eighteen (18) patients suffering from sleep apnea were selected from subgroup I of the ISRUC dataset and were also used to compare scEDR with the more widely used Max Power EDR method (Section 2.6).

### 2.2 Breathing rate from direct pressure-based flow measurements

The breathing rate was estimated using the pressure-based flow channels. The flow channels show a crest and trough that each corresponds to a single breathing cycle inhalation and exhalation, respectively. The crests throughout time were first identified using the standard MATLAB function “findpeaks”. The instantaneous (crest-crest) interval is the time period between two consecutive crests and gives the duration of a single breathing cycle, hence the instantaneous breathing rate (breath per second) frequency is $f_{BR}=\frac{1}{\left(Crest-Crest\right)\;interval}$ . Breathing rate in the standard units of breaths per minute (bpm) is calculated as, ${BR}_{pressure}=\frac{60}{\left(Crest-Crest\right)\;interval}$ (or in terms of the frequency of breathing), ${BR}_{pressure}=60xf_{BR}$ .

### 2.3 Estimation of the heart rate

Heart rate was estimated using a single ECG lead. Firstly, the R waves were detected and then the R-R interval was calculated. Finally, the heart rate was calculated using the instantaneous (R-R) interval (R to R interval) method where HR $=\frac{60}{\left(R-R\right)\;interval}$ . For the computations, the Fieldtrip toolbox was used in MATLAB. (Oostenveld et al., 2011; MathWorks Inc, 2022).

### 2.4 Time-frequency analysis using morse wavelets

Time-frequency analysis (TFA) is a technique to deconstruct a signal to its frequency contents for predefined time windows. There are various TFA methods available however, this study utilized the generalized Morse wavelets as described in (Lilly and Olhede, 2012) due to their flexibility in specifying the wavelet characteristics. Morse wavelets generate a Gaussian wavelet when γ = 3. Meanwhile, the product of β and γ gives the time-bandwidth product. Increasing the time-bandwidth product increases the frequency resolution at the expense of time resolution. Various β parameters were tested to decide on an optimal compromise for the time-frequency uncertainty; it was decided that the best results were given for γ = 3 and β = 90 (best compromise for the time-frequency uncertainty). In this study, calculations were performed using MATLAB with the Lilly and Olhede library (http://www.jmlilly.net/jmlsoft.html).

### 2.5 Reconstruction of breathing signal: the spectral continuity EDR method (scEDR)

**FIGURE 1:**
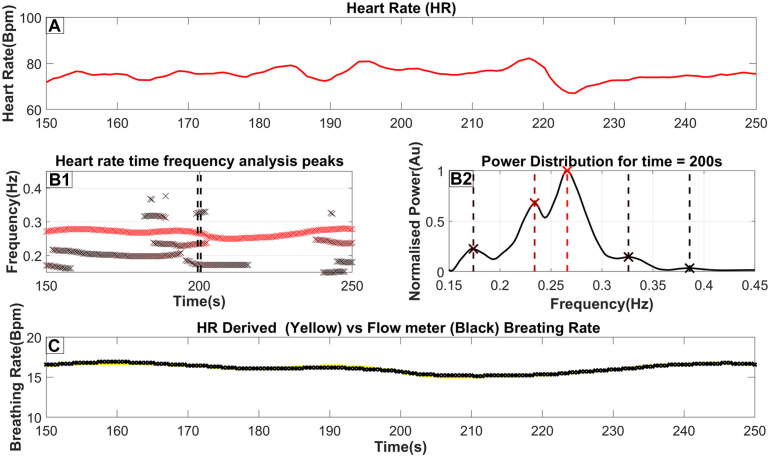
The steps to extract the breathing rate from the ECG (Heart Rate). **(A)** The Heart rate as it is derived from the ECG (as described in Section 2.3). **(B1)** an ‘x’ marks the frequency and time of each peak of the TFA (as described in Section 2.4). Furthermore, the color of the marker indicates the relative power of the peak in that time slice, where a darker red coloring corresponds to a higher power. A threshold of 20% of the time-slices maximum power is used to reject insignificant peaks; however, in this figure, we included all identified peaks for completeness. **(B2)** The power distribution for time = 200s which is also highlighted by 2 dash vertical black lines in **(B1)**. An ‘x’ marker and a vertical dash line indicate the exact peak frequency with its color matching the color seen in part B2. **(C)** The breathing rate for this selected time window with the yellow line indicating the breathing rate extracted from the Heart rate while the black line indicates the breathing rate measured from the flow channel.

TFA analysis was applied as described in Section 2.4 to the heart rate (HR) derived from the ECG measurement (Section 2.3). The frequency in the range of 0.15–0.45 Hz was extracted using a frequency step of 0.02 Hz and a time step of 50 ms (TFA time–slice). For each time slice (i.e., for every 50 ms) the peaks from the spectral power distribution are extracted. A threshold of 20% of the maximum power in each TFA time slice is used to select the significant peaks across the whole frequency spectrum. Then the selected peaks of all TFA time-slices are searched to identify adjacent (in time) peaks of similar frequency that eventually form continuous long-lasting tracks. scEDR selects the longest track; ergo, the frequency of the peak across each TFA time slice is the breathing frequency. The estimated breathing rate is given by the multiplication of the extracted breathing frequency with sixty (60) in the unit’s breaths per minute. We denote this sequence of time-specific breathing rate extracted from the HR as ${BR}_{scEDR}$ . Within the band associated with the respiratory effort, usually, only one track maintains smooth continuity over prolonged periods of time. Other traces of frequency peaks emerge and dissolve as transient short-lived tracks in the time-frequency plane. In cases where the respiratory component is absent (apnea), scEDR selects a candidate track that could join with the previously valid breathing track. A visual representation of the method is illustrated in Figure 1 and is described in Section 3.1 of the results.

By using the results of the method and utilizing the existing TFA analysis results, a signal that is a good estimation of the real breathing pattern can be reconstructed. This is done by applying the inverse Morse-wavelet function on the time-specific TFA values of ${BR}_{scEDR}$ with a frequency window of ±0.015Hz .

### 2.6 Maximum power EDR method

Maximum power EDR uses a similar method to the newly proposed scEDR but instead of forming connections between adjacent peaks across time and then selecting the longest track, it rather selects the peak of highest power as the breathing frequency (see a visual example in the first Figure of section 3.1). In Section 3.2 the Maximum Power EDR is used as a reference to compare the accuracy of scEDR.

### 2.7 Calculating the mean error

The mean error gives a measure of the accuracy of the method that extracts the breathing rate from the heart rate. The percentage error is computed for the measured breathing rate relative to pressure flow meters. The breathing rates are first averaged over thirty (30) seconds corresponding to each sleep stage. The absolute distance between the two rates gives the standard error in breaths per minute. Dividing the standard error by baseline value and multiplying the result by 100 gives the percentage error.

$$\%Error=100\times \frac{\left({BR}_{EDR}-\;{BR}_{Pressure}\right)}{{BR}_{Pressure}}$$

Where, ${BR}_{Pressure}$ is defined as the breathing rate as calculated from the pressure-based sensors and ${BR}_{EDR}$ is the breathing rate extracted from the new scEDR, method.

### 2.8 Appending REM0 definition to established sleep staging

The original sleep staging of human experts was updated according to the automatic reassignment of stages to include the putative sleep stage REM0, as described in (Ioannides et al., 2022). In summary, this procedure uses the variability of the heart rate (HRV) and Global Field Power (GFP) during the eyes closed period before sleep onset to define corresponding thresholds for the variability of HR and GFP. Periods with HR variability lower than its threshold maintain the assignment given to them by the human experts (irrespective of the GFP variability). Periods with HR variability above its threshold are reassigned according to the variability of the GFP: periods of higher GFP variability than its threshold are reclassified as REM0 periods while periods of lower GFP variability than its threshold are reclassified as REM.

## 3 Results

### 3.1 Extraction of breathing rate from heart rate

Part A of Figure 1 shows a heart rate signal in 100-s periods on which TFA was performed. By performing the procedure described above, a power distribution of power across frequency at each time slice is produced as seen in part B2 of Figure 1. The peaks of that power distribution are then localized and shown as ‘x’’s on the same figure. By combining the peaks in many consecutive time slices, part B1 is then produced. These combined peaks assist in utilizing predefined criteria for the longest-lasting continuous sequence which is selected as the respiratory component. Additionally, a threshold derived from the wider spectrum (0.05–0.45 Hz) is used to eliminate insignificant peaks in the 0.15–0.45 Hz band. From this threshold, the breathing rate can then be estimated by essentially monitoring the RSA. Part C shows the Breathing rate (BR) extracted from the Heart rate using a yellow line superimposed on the breathing rate monitored from a real-time pressure flow channel shown with a black line. This highlights the proximity of the estimated BR against the one measured from the flow meters. For this specific section of data, a maximum error of 0.2 breaths per minute was observed. The key observations from the results of Figure 1 are that the BR extracted from the scEDR analysis varies smoothly (Figures 1B1, 1C) and is in excellent agreement with the flow meter results (Figure 1C), even during periods where other frequencies close to the current breathing frequency are present. The complete cross-subject statistics are summarized in Table 2.

**TABLE 2:** The error and %error as explained in Section 2.7 comparing the Max Power method for extracting breathing rate that selects the peak with the highest power compared to the new method described in this paper. The data are derived across the ten (10) healthy subjects using in total 8883 30-s sleep stage periods. Results are broken down to the classical sleep stages as marked by the ISRUC sleep experts. One standard deviation is shown after the ± sign to show the consistency of the results. For each entry the better result (lower error or percentage error) between the Max Power and scEDR is shown with bold numbers.

Sleep stage	Eyes closed awake	NREM1	NREM2	NREM3	REM	Whole night
**Error (Bpm)** **Max power**	1.57 ± 2.52	1.40 ± 2.24	1.29 ± 2.55	0.88 ± 2.23	1.44 ± 2.11	1.29 ± 2.39
**Error (Bpm) scEDR**	**1.17 ± 1.65**	**1.02 ± 1.56**	**0.57 ± 1.09**	**0.24 ± 0.53**	**1.13 ± 1.61**	**0.75 ± 1.33**
**%Error (%)** **Max power**	10.70 ± 18.1	9.65 ± 16.6	9.09 ± 19.1	6.37 ± 17.6	9.45 ± 14.9	8.92 ± 17.79
**%Error (%) scEDR**	**7.51 ± 11.06**	**6.54 ± 9.98**	**3.60 ± 6.96**	**1.49 ± 3.11**	**7.13 ± 10.46**	**4.77 ± 8.66**

### 3.2 Extracted breathing rate and its error across whole night sleep

**FIGURE 2:**
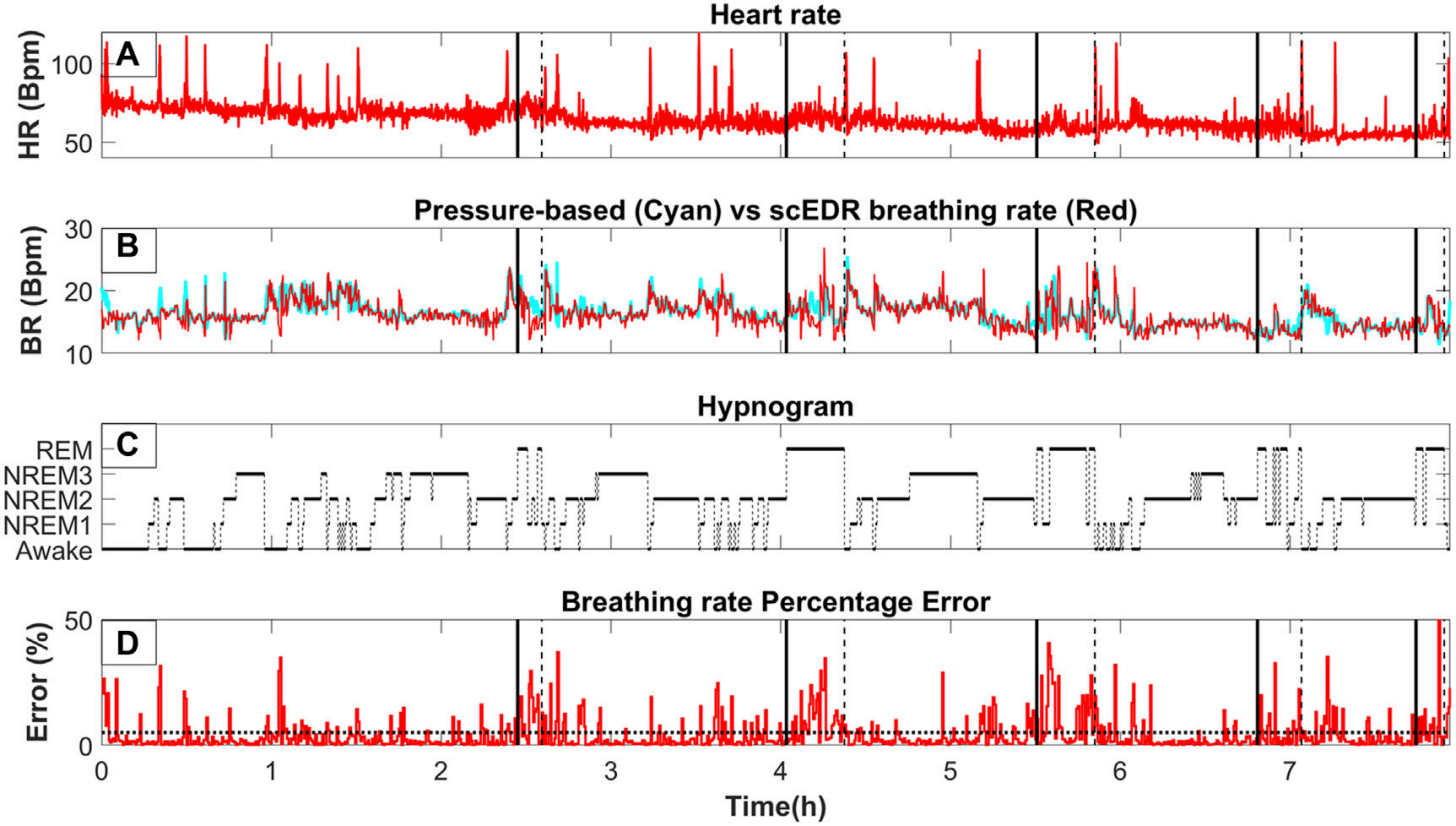
The variation of key measures across whole night sleep; **(A)** The moving average of the heart rate (HR) using a two (2) second window. **(B)** ${BR}_{\text{pressure}}$ (cyan line) and ${BR}_{scEDR}$ (red line) after the same moving average is applied as for the HR in **(A)**. **(C)** the hypnogram as determined by sleep experts. Vertical black lines are used to show the boundaries and mark the transition to and away from REM (as marked by experts). A continuous (solid) black line connects the level of a pre-REM sleep stage (hence, the onset boundary of a REM period) while a dotted black line connects a REM level to its post-REM sleep stage (hence, the offset boundary of a REM period). **(D)** The percentage error in the estimate of BR by our method ( ${BR}_{EDR}$ ) relative to the gold standard ( ${BR}_{\text{pressure}}$ ) as described in section 2.7. The boundaries of REM periods (as defined by the human experts) are transferred from row C to the other three rows **(A, B, D)** as vertical black lines using the same convention (solid and dotted lines marking the onset and offset of REM periods defined by human sleep experts).

Figure 2 shows a whole night recording for a few key metrics of this study for a control subject. On the top row (Part A), the instantaneous R-R heart rate is shown, in the second row (Part B) the extracted breathing rate (Red) and the breathing rate as measured from pressure-based flow meters are shown (Cyan). On the third row (Part C), the hypnogram is displayed while the bottom row (Part D) shows the percentage error between the measured and the extracted breathing rate for each thirty (30) second interval. As can be seen by comparing part A and B of Figure 2, any abrupt increases in the heart rate are also reflected in both the extracted and measured breathing rate. A direct computation of the interaction of the HR and BR could probe whether this correlation is due to an interaction between the two systems, e.g., caused by arousal due to internal or environmental disturbance. However, this effect could also be due to artifacts caused by movement of the body or some electronic noise in the recording system ergo, an artifact captured in both pressure flow and ECG channels. This, however, is beyond the scope of this work and it will be the goal of a future study. The same spikes can also be seen in part D of Figure 2 where the percentage error is displayed. Noisy trials like the ones generating the spikes were not removed even though they are likely to be caused by body movement to test the robustness of the scEDR method during unexpected events and assess whether it can still produce useful estimates of the BR. On part D, a dash horizontal line is drawn at the five (5) % error mark. Most of the percentage error of the trials falls below the 5% error mark (marked by the horizontal dotted line). Large deviations are seen only during and around Rapid Eye Movement (REM) sleep periods with the start and end (Section 2.8) delineated by a solid and dash vertical lines respectively.

### 3.3 Accuracy of results across sleep stages

The robustness of the results of the new method scEDR (as described in Section 2.5) was tested against the previously patented EDR method, which uses the Max Power to select the breathing frequency (Strachan Iain Guy David, 2015) also described in (Bailon et al., 2006) and in section 2.6. The Max Power EDR is a more widely used spectral EDR technique that accepts the peak in the HR frequency analysis with the strongest power as the breathing frequency for that time slice. Table 2 shows the percentage error for both the novel scEDR and the more commonly used Max Power techniques to contrast the accuracy of these spectral EDR techniques that both only utilize the HR and not ECG signal as input. The results of other spectral EDR methods are comparable to the Max Power method (see Table 3 of (Alikhani et al., 2018)). The comparisons described in Section 2.7 are used to compute the error and percentage error for each method (scEDR and Max power). In each comparison, the results of each method are compared against the results obtained with the gold standard measurement (flow meter). The error across all sleep trials (8883) was performed every 30 seconds and then compiled per sleep stage. The comparison of these two methods demonstrates that the introduction of the frequency continuity criterion improves considerably the scEDR accuracy of the breathing rate compared to that of the Max power method. Table 2 outlines the results of this comparison presenting both the error and percentage error. The percentage error calculated for each sleep stage with scEDR showed a markedly decreased value and lower standard deviation (SD), which was particularly prominent in the NREM3 sleep stage (Table 2). The more consistent errors substantiate the grounds for a significantly refined analysis, mitigating uncertainties and bolstering the reliability and credibility of the measurements. Notably, Eyes Closed Awake (ECW), REM and NREM1 display the most pronounced percentage error out of all the sleep stages and the pre-sleep periods, with the lowest error recorded during the NREM2 and NREM3 sleep stages. This is shown for each one of the two methods, thus validating the previous remark based on visual observation of Figure 2. scEDR shows an increased error during ECW and REM that might hinder its ability to accurately monitor breathing during those stages, but it nevertheless outperforms the more widely used Max power EDR through all the stages of sleep. The forthcoming sections of this paper will delve into a comprehensive analysis of the factors contributing to the reduced error and SD resulting from the application of scEDR methodology.

### 3.4 REM error and evidence for the putative REM0 sleep stage

The breathing rate can increase during REM but also, and maybe most importantly, it loses the stability and regularity seen during non-REM (NREM) periods, i.e., between the REM periods. In general, the method seems to work best in between REM periods where the error is the smallest and the cyan and red lines (Figure 2) are almost adjacent to each other. During REM periods, the extracted and measured breathing rates start to separate. Similar breathing patterns, lasting for periods that are comparable to the duration of a continuous sleep stage, are also seen in between REM periods where HRV should not be prominent according to previous studies done in cats (Raetz et al., 1991). These periods are classified by sleep experts mostly as NREM2 and NREM3. The prominent and abrupt changes in breathing and heart rate neither match what is expected during NREM periods nor delineate transitions between NREM sleep stages. According to the previous results from Figure 2 and Table 2, most errors arise around and during the REM sleep stage. To investigate possible correlates of this high error rate the spectra of 90-second-long periods from each sleep stage are extracted, including REM0. The procedure of reassigning sleep stages to include REM0 is described in Section 2.8. A similar table with Table 2 of this manuscript can be found in Supplementary Table S1 containing the same comparison for the sleep apnea patients while removing periods marked as sleep apnea or hypopnea by the sleep experts.

**FIGURE 3:**
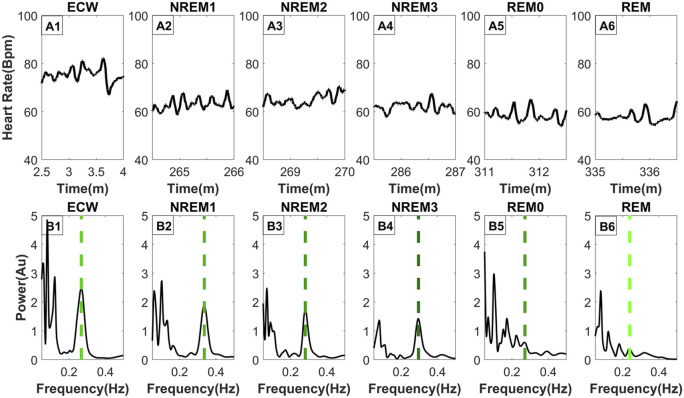
Frequency distribution of the heart rate across typical sleep stage periods. The figure has two rows, the top row [Parts **(A1–A6)**] shows the heart rate across time for ninety (90) seconds, while the bottom row [Parts **(B1–B6)**] shows the frequency distribution using morse wavelets for the above heart rate series. The placement of the dashed line indicates the peak corresponding to the breathing frequency, while its color defines the LF/HF ratio (the lighter shade of green indicates a higher LF/HF ratio). All-time series displayed (top row) were selected from the same subject and all from the third sleep cycle except ECW which was selected at the start of the experiment.

The top row of Figure 3 (Parts A1, A2, A3, A4, A5, A6) shows the HR for the representative segments of each sleep stage, including REM0. The spectrum of the HR of each panel in the top row is computed and displayed directly below (Parts B1, B2, B3, B4, B5, B6). The percentage (across subject) of REM0 previously classified (by the sleep experts) in the original sleep scoring was: 16.9% ECW, 15.2% NREM1, 31.7% NREM2, 28.1% NREM3 and 8.1% REM. Additionally, a longer period contrasting the differences in the morphology of the HR between REM0, and REM can be seen in Supplementary Figure S1. The displayed spectra (Figures 3B1–B6) show that during the ECW, NREM1, NREM2 and NREM3 sleep stages, there is a very prominent peak in the High Frequency (0.15–0.40 Hz) band (HF) that corresponds to the breathing component. Furthermore, this well-separated breathing peak is the major contributor to the HF band’s total power. In contrast, in the REM and REM0 stages, many peaks in the HF band are of similar or even higher strength, therefore there is more than one component, making significant contributions to the HF band’s total power; the presence of the other peaks makes it difficult for Max Power EDR method to select the actual breathing frequency. These observations explain why during the REM and REM0 stages, the breathing component is hard to select if the only criterion is the peak strength and provide further support for the introduction of REM0 as a distinct sleep stage.

### 3.5 Spectral heart rate variability across sleep stages

**FIGURE 4:**
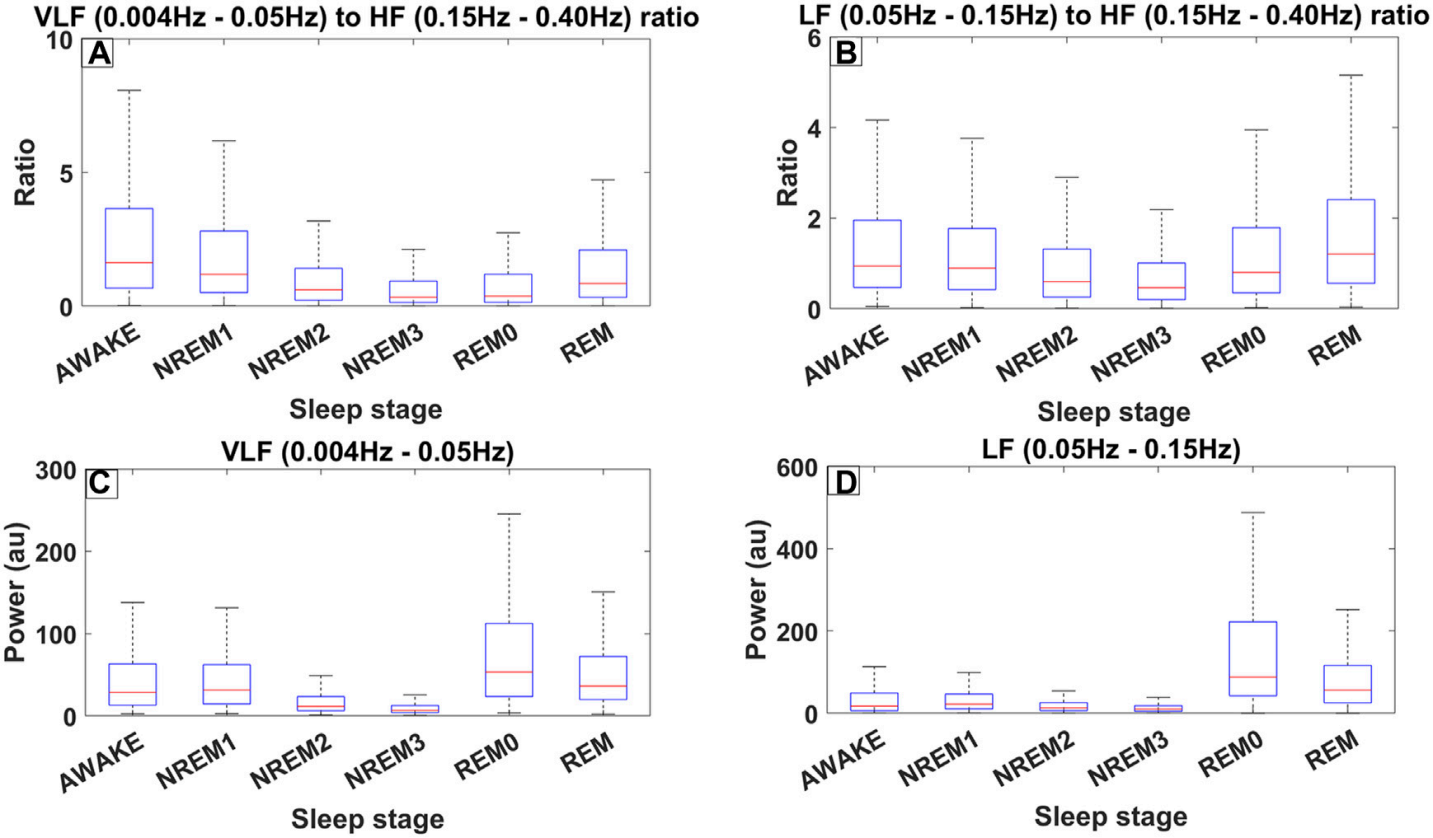
Different measures of spectral heart variability across all trials (8889) for all 10 subjects of ISRUC subgroup III separated across sleep stages including REM0. The frequency bands used were defined according to the accepted standards (Shaffer and Ginsberg, 2017). **(A)** Boxplots across different sleep stages for the ratio between the Very Low Frequency (VLF) band (0.004–0.05 Hz) and High Frequency (HF) band (0.15–0.40 Hz). **(B)** Boxplots for the ratio between the Low Frequency (LF) band (0.05–0.15 Hz) and HF. **(C)** Boxplots illustrating the power in the VLF band. **(D)** Boxplots illustrating the power in LF band.

Figure 4 shows the distribution of the LF/HF ratio and the VLF/HF ratio separated per sleep stage across all 8893 30-s sleep trials. Both ratios decrease from the awake state to the NREM3 sleep stage, and they then increase again in the REM0 and REM stages where they reach similar levels to that of the awake stage (Figures 4A, B). From the previous arguments, it can be inferred that the parasympathetic system gains increased influence as sleep progresses to the NREM3 stage until the sympathetic system retakes control during REM sleep stages. Interestingly, an inspection at the bottom boxplots of Figure 4 (Figures 4C, D), where the distribution of the power in the LF and VLF bands are shown, reveals that the LF power for both REM and REM0 sleep stages is higher than that in the awake state. Meanwhile, for the VLF band, REM has a similar power to that of the awake state while REM0 reaches a higher spectral power than the awake state.

## 4 Discussion

### 4.1 Advantages of spectral continuity EDR (scEDR)

EDR, i.e., extracting the breathing rate (BR) from ECG was first introduced in the 1980s (Moody, 2008). Since then, the field has evolved into two main classes of methods: the first set of methods used the morphology of the ECG signal and the second class employed the spectral decomposition of either the ECG or the Heart rate (Sohrt-Petersen, 2014). Although methods that extract the breathing rate from the heart rate are often clumped together into the wider EDR methods family, this might be misleading as Heart rate can often be derived from methods other than ECG such as directly from the heart rate derived from photoplethysmography (PPG). Some of these measures are often simpler and cheaper to implement in daily monitoring such as PPG, nowadays present in many smart wristwatches (Ghamari et al., 2018). Therefore, to make breathing monitoring as widely available as possible, this study focused on frequency-based EDR methods that only require the heart rate and not the ECG signal and how we can improve them. Although, scEDR, has shown an increased accuracy against the more widely used Max Power EDR and achieves an error of less than 5% for the whole sleep, scEDR has some limitations particularly during ECW, NREM1 and REM stages where the percentage error against the flow meters is between 6.5% and 7.5%. Furthemore, this study has only performed scEDR on sleep data and therefore requires further testing to ensure scEDR’s accuracy during the awake and high intensity exercise states.

Previous studies have already used discrete wavelet transformation to extract the breathing signal from the heart rate or ECG signal (Yi and Park, 2002; Zhao et al., 2008). This study takes a different approach to extract breathing. Instead of extracting the breathing signal and then calculating the breathing rate, the specific frequency components corresponding to respiration is firstly identified and subsequently, the inverse Morse wavelet transform is applied to reconstruct the breathing signal in a small frequency window. By doing so, this method avoids using the whole HF band (0.15–0.40 Hz) to reconstruct the breathing signal as it is often thought to be the “cardiac parasympathetic modulation” (Hedman et al., 1995) and not just the breathing component. Furthermore, older methods used a peak detection algorithm to just select the strongest frequency (Max power in section 2.6) within the HF band based on the assumption that the breathing frequency is the major contributor to the HF band (Strachan Iain Guy David, 2015). As a consequence, spurious abrupt changes in BR can emerge. These are due to components not related to breathing but characterized by similar frequencies, becoming briefly stronger than the true BR and hence misidentified as the BR. The new method, scEDR, avoids the problem by imposing a continuity constraint and a dynamic threshold (see section 2.5). Most of the time, it is possible to extract a single continuous BR estimate. The litmus test supporting the validity of the method is that the continuous smooth scEDR-reconstructed estimate of BR coincides with the gold standard BR measured from the flow sensors, as demonstrated in the displays of Figure 1.

The holy grail of measuring any quantity, especially when related to medicine and human subjects, is to be able to measure accurately, in real time and with the least discomfort to the patient. Current flow meters provide high accuracy and real-time data, nevertheless, they use invasive and expensive equipment. A recent study was able to extract breathing rate non-invasively by relating the PPG signal’s envelope modulations with respiratory activity (Sultan and Saadeh, 2022). However, they concluded that they could not monitor breathing activity continuously and required further research to improve their method’s robustness. Achieving the goal of reliable and widely available real-time pulmonary monitoring requires triple capability: 1) extraction of the breathing rate 2) extraction of a breathing analogue signal and 3) performing the two extractions in almost real-time without the need for a large volume of data (given a small buffer window to avoid the edge effect). The proposed method, scEDR, attempts to deliver this seemingly impossible triad. This is based on the ability to extract the smooth continuous record of respiration from the heart rate, i.e., Respiratory Sinus Arrhythmia (RSA). The success of scEDR implies that the RSA persists and remains smooth, at least in healthy subjects during awake state and sleep, although we have not yet tested the awake state, except for the ECW part of the ISRUC data. There are indications from our preliminary investigations that this may also be the case during episodes of sleep apnea, but addressing this in more detail to make a statement about future clinical uses requires more research and is beyond the scope of this work. As can be seen in the didactic work of Section 3.1 and illustrated in the visual representation of the method shown in Figure 1, the continuity constraint endows scEDR with the capability of accurately monitoring RSA even in periods with other artifacts introducing components with frequencies close to the breathing frequency. In summary, despite some promising findings in our limited work so far, more studies must be performed to allow a good evaluation of its potential, particularly on a wider range of age groups including elderly and teenage subjects. An accurate extraction of breathing patterns for elderly patients may have useful clinical applications however, studies have shown a weaker RSA pattern in old age (Masi et al., 2007). Therefore, even though due to scEDR’s continuity filter enhanced detection of RSA was shown, future studies may be required that focus on applying scEDR specifically on elderly patients.

### 4.2 Spectral heart rate variability across sleep stages

Previous studies have suggested that the Low Frequency/High Frequency (LF/HF) ratio of the heart rate spectral decomposition can be used as an indicator for the autonomic balance (Task Force of the European Society of Cardiology and the North American Society of Pacing and Electrophysiology, 1996) and that increases in the VLF (0.004–0.05 Hz) band are associated with a higher risk of morbidity (Wang et al., 2012). To that effect, LF activity (0.05–0.15 Hz) is thought to be increased by the sympathetic nervous system while, HF activity (0.15–0.40 Hz) is mostly affected by the parasympathetic system even though our results show that the breathing rate is a major contributor to this band. Hence, a high LF/HF activity means that the sympathetic system is currently in control while a low LF/HF indicates that the effects of the parasympathetic system predominate.

The results described in sections 3.4 and 3.5 and presented in Figures 3, 4 show that during sleep the (LF/HF and VLF/HF) ratios vary extensively in healthy subjects. While in awake state and all sleep stages except REM (and the putative REM0 period) these ratios are largely determined by a dominant peak at high frequencies, well above 0.2 Hz, which is greatly reduced for REM and REM0. Given the role of these ratios for the autonomic balance and particularly the putative association of increases of the VLF (0.004–0.05 Hz) band with a higher risk of morbidity, there is an urgent need to disentangle the mechanisms involved in the variations we have observed during normal sleep and study them in detail in pathology, both during sleep and awake state.

### 4.3 REM0 implications

In previous studies, REM0 periods were defined based on distinctive patterns in the HR. Initially, these observations related to surges in HR in periods when PGO waves were encountered in cats (Rowe et al., 1999). In the end, the definition of REM0 relied on HR variability, which has a clear-cut definition and algorithmic implementation. Under this definition periods of light, deep sleep and REM are re-classified under the putative REM0 sleep stage (Ioannides et al., 2022). The EEG patterns encountered during REM0 appeared as anomalies in the hypnogram derived from the classical 7-stage sleep classification (Rechtschaffen and Kales, 1968). These anomalies contained adjacent periods with features characterizing distinct sleep stages and consequently, they corresponded to periods when the sleep staging rules were most difficult to apply. Consequently, these anomalies appeared where sleep experts usually disagreed on the sleep staging definition. The latest amendments (Iber, 2007; Silber et al., 2007) are an attempt to force uniformity in sleep scoring. The new rules for sleep staging simply forced a choice of one or other sleep stage by defining thresholds of the percentages of grapho-elements belonging to one or other of the classical sleep stages and the amalgamation of NREM3 and NREM4 into a single sleep stage for deep (or slow wave) sleep, the new NREM3 sleep stage (Silber et al., 2007). The old anomalies were hidden by these definitions, but careful inspection of the resulting hypnogram revealed important physiological changes in the middle of one continuous sleep stage (Ioannides et al., 2022).

From the results of this study seen in Sections 3.2, 3.3, most of the errors from the max Power and the scEDR methods arise during and around REM. The reason can be seen in Section 3.4 where typical spectral maps extracted from the HR are shown for each sleep stage. In contrast to the other sleep stages REM and REM0 stand out as the only sleep stages where there are multiple prominent peaks in the HF band. According to classical sleep staging during REM sleep, the EEG and Magnetoencephalography (MEG) closely resemble that of the awake state mostly in the beta and gamma frequency band (Cavelli et al., 2017; Simor et al., 2020) while during NREM sleep brain activity is dominated by the low frequencies (Dijk, 1995; Nir et al., 2011). REM0 poses an interesting intermediate sleep stage where a unique combination of features are present. The EEG and MEG power is dominated by the lower frequencies most closely resembling NREM2 and NREM3; nevertheless, at the times of the surges, the low-frequency carriers of the multiple K-complexes (KCm) coexist with strong high-frequency power (beta and gamma bands), which however have a smaller impact in the overall wide-band power, as seen in Figure 3 of this study (Ioannides et al., 2022). Also, the heart rate and breathing rate pattern most closely resemble those patterns in REM sleep. These observations enhance the claim of REM0 as a putative distinct sleep stage that was suggested in a previous publication (Ioannides et al., 2022).

Sleep normally progresses in sleep cycles of around ninety (90) minutes each going from awake or NREM1 to NREM3 and finally to the REM stage completing the cycle. During each sleep cycle changes are encountered in both the EEG and MEG brain activity (as described above) and in the physiological state as shown in section 3.5. Specifically, the LF/HF and VLF/HF exhibit a “u-shape” curve between Awake and REM, as seen in Figures 3A, B. If assume that LF/HF is positively correlated with sympathetic activity, we can infer that in REM sleep and the awake state, the sympathetic dominance is at similar levels. Furthermore, sympathetic activity drops from awake as sleep progresses to reach its lowest levels during NREM3 and then rebounds to awake-like levels through the passage from REM0. These findings are in line with previous studies that described a similar transition through sleep (Trinder et al., 2001; Sumi and Kadotani, 2022). We conclude that the analysis with REM0 provides tentative support for the variation of the autonomic balance in accordance with the cited literature in this paragraph.

Previous animal experiments have shown that Adenosine Triphosphate (ATP) production is strongly associated with delta waves during NREM sleep especially in the first sleep cycles (Dworak et al., 2010) where coincidentally, REM0 was also found to be at its longest (Ioannides et al., 2022). Additionally, low ATP levels were found to increase the duration of NREM sleep (Kalinchuk et al., 2003) and a higher AMP/ATP ratio produces stronger “EEG Slow wave activity (0.5–4 Hz)” during NREM sleep (Chikahisa and Séi, 2011). REM0 is in an interesting position where it has both slow wave activity in the form of K-complex multiples (KCm), but KCm events with increased gamma band activity are found during heart rate surges. Similar heart rate surges are also present during REM sleep, nonetheless without any slow wave activity or large grapho-element in the EEG or MEG. These surges are likely linked with the increase in the LF and VLF bands seen during those periods (Figure 4). The increase in the LF and VLF bands between REM0 and the NREM stages further cements its position as an independent sleep stage. Therefore, we suggest that REM0 might serve as a middle ground to prepare the body for the energy-intense functions of REM (Ioannides et al., 2009; Ioannides, 2018).

The current sleep classification system is mostly based on the so-called hallmarks of sleep, large EEG/MEG grapho-elements (Colten et al., 2006) mostly ignoring other physiological measures such as the Heart rate. Even when muscle tone and eye movements are mentioned in the manual they mostly serve as a verification of the sleep stage and not as an indicator. Our results further strengthen the case for REM0 inclusion in the sleep staging definition; however, we do not currently have enough data to argue whether REM0 should be included in the existing mostly EEG/MEG-based classification or whether another set of sleep stage classifications based on physiological indicators should be introduced to include REM0 like epochs. A similar dual system classification exists when classifying the menstrual cycle which consists of two overlapping cycles the ovarian and the uterine cycle (Haroun, 2016; Thiyagarajan et al., 2022). Similarly, if a two-system sleep classification system were introduced the existing classification mostly based on the hallmarks of sleep would be kept, while a new classification system would be introduced that considers the physiological indicators of heart rate, breathing rate, muscle tone, eye movement but further include other important but overlooked measures, e.g., galvanic skin resistance (GSR).

### 4.4 Real-life applications and clinical significance

Currently, there is a gap in the medical setting where an accurate yet low-cost method for measuring the breathing of a patient does not exist. Previous EDR techniques utilized mostly the ECG, avoiding the use of HR because of the intrusion of other non-breathing components. The scEDR method avoids this problem, as extensively described earlier, allowing accurate breathing rate estimates from the HR. Therefore, an argument can be made for implementing our method alongside pulse oximeters, commonly used in PPG studies by making use of new powerful microchips. Such a combination could provide, using a single and widely available clinical sensor, the breathing rate in addition to the heart rate and blood saturation already provided routinely with the pulse oximeter. An outcome of this integration would be a new class of pulse oximeters that measure both cardiac and pulmonary components. Such data could serve as the basis for future studies to validate scEDR’s accuracy in the clinical setting as this study has only validated scEDR’s efficacy using sleep data of healthy subjects. An outlook arising for a future study would be to examine whether there is a correlation between the tidal volume, i.e., the wave height from mass flow meters and the wave height from the reconstructed breathing component. Previous studies have already investigated the relationship between tidal volume and RSA and found a multifactorial relationship. In this study, the authors found that increasing the breathing frequency between seven (7) and forty (40) breaths per minute shows a decrease in the RSA while oxygen content also affects the strength of RSA. Nevertheless, the authors established a strong positive linear correlation between tidal volume and RSA when controlling the breathing frequency (Hirsch and Bishop, 1981).

Sleep apnea is a condition that is currently mostly undiagnosed in the population (Heinzer et al., 2015), but is nevertheless associated with an increased risk of many comorbidities (Schreib et al., 2020; Kalaydzhiev et al., 2023). Attempts to automatically detect sleep apnea have been made in the last years either using deep learning from ECG data (Zhang et al., 2021; Kumar Tyagi and Agrawal, 2023) or even by detecting differences in the frequency power spectrum of EEG (Saha et al., 2019). Alongside the development of these techniques, there has been an unprecedented rise in the use of smartwatches, a lot of which are being advertised as health monitoring devices. A common feature is the inclusion of PPG sensors utilized as a blood oximeter that provide many health indices including the heart rate (Massoomi and Handberg, 2019). Smartwatches are already being assessed for their use in monitoring patients with atrial arrhythmia (Bumgarner et al., 2018; Koshy et al., 2018). Our proposed method in contrast with existing methods performs well with just the heart rate (available on most smartwatches) and does not require the ECG signal. Therefore, a new application would be to integrate the methods described in Section 2.5 with smartwatches that have built-in PPG sensors. This would be a tremendous advancement in decreasing the prevalence of undiagnosed sleep apnea.

### 4.5 Conclusion

By taking a different approach to ECG Derived Respiration, this study utilized the heart rate in contrast to ECG signal morphology as our primary metric. It subsequently performed a time-frequency analysis on the heart rate and selected long-lasting spectral components to identify the most probable peak frequency corresponding to the RSA. This approach allows the extraction of breathing rate from just pulse oximeters thus making scEDR more friendly towards a clinical setting if future studies validate its accuracy using awake and exercise data. The validation test of the extracted breathing rate for 10 healthy subjects with whole-night sleep using scEDR yielded an error of 4.77% (8.92% when using the max power method) against the pressure flow meter-derived respiration. By observing that most of the error was focused during and near REM periods, this study investigated the power distribution of the heart rate during typical sleep stages, considering the putative REM0 sleep stage. This revealed an increase in the number of spectral components present in the High-frequency band (0.15–0.40 Hz) only during REM and REM0. However, these high-frequency components were markedly lower in terms of power than the single clear high-frequency peak encountered in awake state and all other sleep stages. The Heart rate spectral distribution shows REM0 to be closer to REM even though using classical sleep staging (EEG) it would be often classified as NREM2 or NREM3 and even NREM1 and REM in some cases.

## Data Availability

The original contributions presented in the study are included in the article/Supplementary Material, further inquiries can be directed to the corresponding author (g.orphanides@aaiscs.com) or senior author (a.ioannides@aaiscs.com). All data used in this study are open-access data from the ISRUC-SLEEP dataset available at: https://sleeptight.isr.uc.pt/.
