# Supplementary material for: Using spectral continuity to extract breathing rate from heart rate and its applications in sleep physiology
Source: Front Physiol. 2024 Aug 2;15:1446868. doi: 10.3389/fphys.2024.1446868 (PMC11327063; doi:10.3389/fphys.2024.1446868)
Supplement: Supplementary file 1 [file DataSheet1.PDF]

Supplementary Material

1 Supplementary Figures and Tables

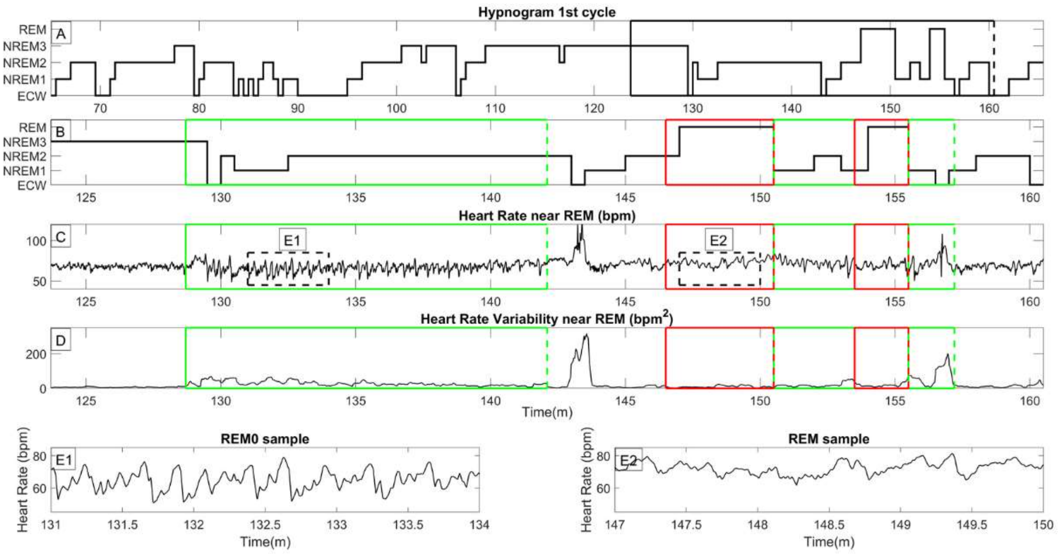

Supplementary Figure 1: Differences in the Heart rate rhythm between REM and the putative REM0 sleep stage. A) Hypnogram for first sleep cycle of a healthy subject as classified by the sleep experts. The black boxes mark the period selected for parts (B), (C) and (D). B) Zoomed segment of the hypnogram of part (A) showing the sleep experts' classification (black) and the augmented definitions with REM0 (green) and REM (red roughly agreeing with the experts' classification). C) Heart rate shown for the cut-out segment of part (B), keeping the green and red boxes marking the REM0 and REM boundaries. In both parts (B) and (C) the display starts 5 minutes before the onset of REM0 and ends 5 minutes after the offset of the last REM period. The boxes defining the cut-out section in part (A) and the reclassified periods as REM0 (green) and REM (red) mark the start with a dash vertical side. The dash black boxes indicate the periods that are shown in parts E1 and E2. D) Heart rate variability shown for the same time period as in (B) and (C). E1) Close up section of a 3-minute period of REM0 . E2) close up section of a 3-minute period of REM. The same Heart rate scale is used (E1) and (E2) to aid comparison.

Supplementary Table 1. The error and %error for 18 patients with sleep apnea while removing epochs marked by sleep experts either as sleep apnea or hypopnea. The same format is used as that of Table 1 for easy cross-reference. Results are broken down to the classical sleep stages as marked by the ISRUC sleep experts. One standard deviation is shown after the ± sign to show the consistency of the results. For each entry the better result (lower error or percentage error) between the Max Power and scEDR is shown with bold numbers, showing that for all cases the result of scEDR is always better than the corresponding result of the Max Power method.

Sleep Stage	Eyes Closed Awake	NREM1	NREM2	NREM3	REM	Whole Night
Error (Bpm) Max power	1.39±1.56	1.22±1.45	1.08±1.38	0.78±0.83	1.35±1.56	1.16±1.36
Error (Bpm) scEDR	**1.25±1.60**	**0.99±1.32**	**0.82±1.12**	**0.54±0.83**	**1.07±1.60**	**0.93±1.30**
%Error (%) Max power	9.46±11.15	8.33±10.30	7.55±9.77	5.37±5.84	8.73±10.23	7.89±9.46
%Error (%) scEDR	**8.09±10.27**	**6.58±8.63**	**5.47±7.41**	**3.39±5.41**	**6.76±9.86**	**6.06±8.32**
